# Exercise-Induced Shear Stress Drives mRNA Translation In Vitro

**DOI:** 10.3390/cimb46090589

**Published:** 2024-09-05

**Authors:** Daniel Conde, Mario A. Garcia, Manuel Gomez, Alvaro N. Gurovich

**Affiliations:** 1 Clinical Applied Physiology (CAPh) Lab, The University of Texas at El Paso, El Paso, TX 79968, USA; mgomez26@utep.edu (M.G.); agurovich@utep.edu (A.N.G.); 2 Department of Physical Therapy and Movement Sciences, The University of Texas at El Paso, El Paso, TX 79968, USA; 3 Driskill Graduate Program in Life Sciences, Northwestern University Feinberg School of Medicine, Chicago, IL 60208, USA; mario.garcia@northwestern.edu; 4 Interdisciplinary Health Sciences Ph.D. Program, The University of Texas at El Paso, El Paso, TX 79968, USA

**Keywords:** endothelium, exercise-induced shear stress, eNOS, SIRT1, ET1, NOS3, EDN1

## Abstract

The vascular endothelium is the first line of defense to prevent cardiovascular disease. Its optimal functioning and health are maintained by the interaction of the proteins—endothelial nitric oxide synthase (eNOS), sirtuin 1 (SIRT1), and endothelin 1 (ET1)—and the genes that encode them—*NOS3*, *SIRT1*, and *EDN1*, respectively. Aerobic exercise improves endothelial function by allegedly increasing endothelial shear stress (ESS). However, there are no current data exploring the acute effects of specific exercise-induced ESS intensities on these regulatory proteins and genes that are associated with endothelial function. The purpose of this study was to assess the acute changes in endothelial proteins and gene expression after exposure to low-, moderate-, and high-intensity exercise-induced ESS. Human umbilical vein endothelial cells (HUVECs) were exposed to resting ESS (18 dynes/cm^2^, 60 pulses per minute (PPM)), low ESS (35 dynes/cm^2^, 100 PPM), moderate ESS (50 dynes/cm^2^, 120 PPM), and high ESS (70 dynes/cm^2^, 150 PPM). Protein and gene expression were quantified by fluorescent Western blot and RTqPCR, respectively. All exercise conditions showed an increase in eNOS and SIRT1 expression and a decrease in *NOS3* and *SIRT1* gene expression when compared to resting conditions. In addition, there was no expression of ET1 and an increase in *EDN1* gene expression when compared to resting conditions. These results show that (1) exercise-induced ESS increases the expressions of vascular protective proteins and (2) there is an inverse relationship between the proteins and their encoding genes immediately after exercise-induced ESS, suggesting that exercise has a previously unexplored translational role catalyzing mRNA to proteins.

## 1. Introduction

Cardiovascular disease (CVD) remains the leading cause of death worldwide, accounting for 17.8 million deaths [1,2]. Atherosclerosis, a chronic inflammatory vessel disorder, contributes to developing CVDs, including myocardial infarction and stroke [3]. The progression from atherosclerosis to CVD starts with molecular processes that result in endothelial cell dysfunction that causes damage to the arterial wall, followed by the accumulation of atherosclerotic plaque [4]. A healthy endothelium regulates vascular structure and tone by releasing dilator and constrictor molecules in response to different stimuli including shear stress, which is an essential biomechanical mechanism created by the interaction between cells and fluid flow [5,6].

Endothelial cell shear stress (ESS) is the frictional drag force produced by the blood flow exerted on the luminal surface of vascular endothelial cells [7]. Endothelial cells contain multiple ESS sensors that convert this biomechanical force into a biochemical stimulus. Moreover, endothelial cells detect variations in shear stress magnitude, regulating endothelial cell signaling pathways, cell structure remodeling, and gene expression [8,9,10,11,12,13]. Physical inactivity decreases ESS, inducing cellular apoptosis, an accumulation of reactive oxygen species (ROS), and reducing the enzymatic activity of key molecules associated with proper endothelial function [14,15,16]. Alternatively, exercise-induced ESS creates higher shear stress, acting as a protective mechanism against CVD [17,18,19].

The increasing costs and allocation of resources for treating CVDs [20,21] have encouraged researchers to explore the role of crucial biomolecules involved in the regulation of vascular homeostasis. Arguably, the most important molecule is endothelial nitric oxide synthase (eNOS), encoded by the *NOS3* gene, which is a tightly regulated enzyme that catalyzes the oxidation–reduction reaction for the production of the vasodilator nitric oxide (NO) by using L-arginine and oxygen (O_2_) as substrates [22]. Another key molecule is sirtuin 1 (SIRT1), encoded by the *SIRT1* gene, which is a ubiquitous protein expressed in different tissues, including endothelial cells. Endothelial SIRT1 contributes to the protection of the endothelium by regulating various proteins. In the endothelium, SIRT1 and eNOS have been shown to regulate one another using positive feedback loops [23]. Finally, vasoconstrictor endothelin 1 (ET1), encoded by the *EDN1* gene, is a peptide that increases blood pressure, contributing to vascular damage and leading to CVDs [24]. The expression of these proteins and the genes that encode them has been shown to be associated with the increased ESS produced during exercise [25,26,27,28,29,30].

Although exercise-induced ESS has beneficial effects in improving endothelial cell function, the acute and different exercise intensity-dependent ESS effects are unexplored. Currently, in vitro models investigating the role of ESS on cellular homeostasis have not included a pulsatile flow system and use low-intensity shear stress, which does not reflect the ESS experienced during exercise conditions in vivo. Therefore, this experiment aimed to determine the role of different magnitudes of exercise-induced ESS on the acute expression of proteins associated with endothelial health—eNOS, SIRT1, and ET1—and their associated genes—*NOS3*, *SIRT1*, and *EDN1*.

## 2. Materials and Methods

### 2.1. Cell Culture

Pooled human umbilical vein endothelial cells (HUVECs; Cell Applications, San Diego, CA, USA) were cultured following our previously described methods [31] using MesoEndo cell medium (Cell Applications, San Diego, CA, USA) in a T-75 cell culture flask (Corning Inc., Corning, NY, USA) inside an incubator at 37 °C with 5% carbon dioxide (CO_2_) concentration until they were 80% confluent. Once cells reached 80% confluence, they were trypsinized and seeded in straight-channel µ-slides I Luer (Ibidi USA, Fitchburg, WI, USA) and were incubated at 37 °C with 5% CO_2_ until cells reached 80% confluence. Once cells reached 80% confluence, four different intensities of ESS were applied.

### 2.2. Experimental Shear Stress

The intensity of the exercise-induced ESS was calculated from previous in vivo studies [6,32,33]. Four pulsatile flow intensities were applied for six hours using the Ibidi Pump System (Ibidi USA, Fitchburg, WI, USA) inside an incubator at 37 °C with 5% CO_2_. All the conditions consisted of five hours of resting ESS and one hour of experimental ESS as follows: resting at 18 dynes/cm^2^ with 60 pulses per minute (PPM) for a total of 1080 dynes/cm^2^/min, low shear stress (LSS) at 35 dynes/cm^2^ with 100 PPM for a total of 3500 dynes/cm^2^/min, moderate shear stress (MSS) at 50 dynes/cm^2^ with 120 PPM for a total of 6000 dynes/cm^2^/min, and high shear stress (HSS) at 70 dynes/cm^2^ with 150 PPM for a total of 10,500 dynes/cm^2^/min. All the ESS conditions were randomly repeated and four slides were obtained for each intensity. The stimulated cells were pooled together to ensure an optimal mRNA concentration. The same process was repeated to achieve an optimal protein concentration.

### 2.3. mRNA Extraction and RTqPCR

Following ESS conditions, cells were washed with DPBS (Gibco, Grand Island, New York), harvested, and total mRNA was extracted using the Qiagen RNeasy Micro Kit (Qiagen, Hilden, Germany), following the manufacturer’s instructions. After the mRNA was extracted, the integrity and purity of the samples were confirmed using a NanoDrop 1000 Spectrophotometer (Thermo Scientific, Waltham, MA, USA). The absorbance ratios (A260/A280) were between 1.8 and 2.0, indicating a high purity and minimal contamination. All the samples contained 0.5–1 µg mRNA and were reverse transcribed into complementary DNA (cDNA) using the High-Capacity cDNA Reverse Transcription kit (Applied Biosystems, Waltham, MA, USA) using the GeneAmp PCR System (Applied Biosystems, Waltham, MA, USA). The target genes *NOS3* (Hs01574665_m1), *SIRT1* (Hs1009006_m1), and *EDN1* (Hs00174961_m1), as well as the housekeeping gene *GAPDH* (Hs02758991_g1), were amplified using TaqMan Fast Advanced master mix and probes (Applied Biosystems, Waltham, MA, USA) in duplicate (*n* = 2). Their relative expression was quantified using the StepOne Real-Time PCR System (Applied Biosystems, Waltham, MA, USA) using the ΔΔCt method, subtracting the expression of the housekeeping gene *GAPDH* from the expression of the target genes, and comparing the expression from each experimental condition to the unstimulated control. The relative expression was converted to fold changes using the formula 2^−ΔΔCt^. To summarize the distribution of the fold changes and to simplify the interpretation of the upregulation/downregulation of genes, the fold changes were transformed using the formula log_2_ (fold change).

### 2.4. Protein Extraction and Western Blot

Cells were harvested immediately after exposure to ESS. Cells were washed with Dulbecco’s phosphate-buffered saline (DPBS; Gibco, Grand Island, New York, NY, USA) and were lysed inside the µ-slides using a Cell Lysis Buffer (Cell Signaling, Danvers, MA, USA). Lysed cells were collected and centrifuged at 14,000× *g* for 10 min at 4 °C. The supernatant containing proteins was collected, and total protein content was measured using the bicinchoninic acid (BCA) assay (Thermo Scientific, Waltham, MA, USA). Samples were diluted to a final protein concentration of 15 µg, loaded into a Mini-PROTEAN TGX precast gel (Bio-Rad, Hercules, CA, USA), and separated by molecular weight using electrophoresis. Separated proteins were transferred to a polyvinylidene difluoride membrane (PVDF; Bio-Rad, Hercules, CA, USA), blocked for two hours at room temperature using Intercept blocking buffer (LiCor, Lincoln, NE, USA), and incubated overnight at 4 °C in the target primary antibodies eNOS (Abcam, Cambridge, UK), SIRT1, and ET1 (Santa Cruz Biotech, Dallas, TX, USA), as well as the loading control GAPDH (Abcam, Cambridge, UK). After primary antibody incubation, PVDF membranes were incubated in fluorescent secondary antibodies IRDye 800 and IRDye 680 (LiCor, Lincoln, NE, USA) for two hours at room temperature in the dark. Proteins were detected and quantified using the Licor Odyssey CLx imaging system (Licor, Lincoln, NE, USA), and the loading control protein GAPDH was used to normalize the expression of the target proteins by dividing the target protein concentration by GAPDH concentration. All pooled samples exposed to ESS (*n* = 4) were run in duplicate, and the control (*n* = 4) was run without a technical replicate.

### 2.5. Statistical Analysis

Quantified fluorescence signals from Western blot experiments were analyzed for significant differences between different conditions using the Kruskal–Wallis H test. The analysis was performed using R statistical software (Version 2023.06.0, R Core Team, 2023). Results were considered statistically significant at a *p*-value < 0.05.

## 3. Results

### 3.1. Gene Expression

**Figure 1:**
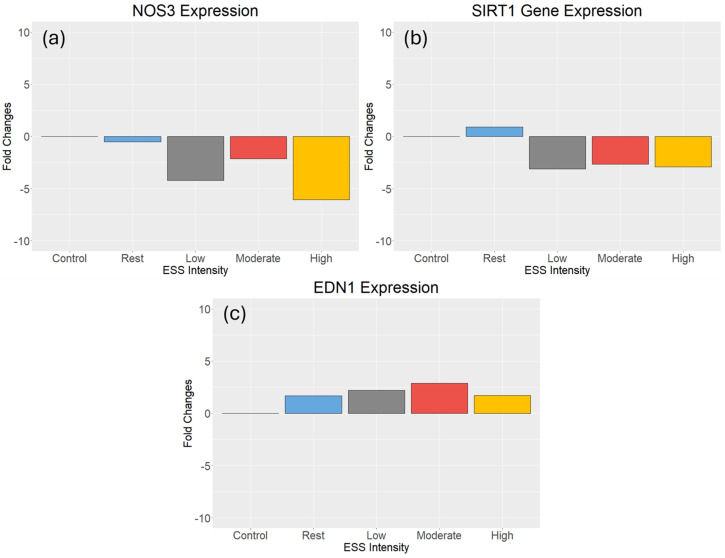
Gene expression after exercise-induced ESS. (**a**) HSS showed the greatest downregulation of *NOS3*, followed by LSS and MSS (*n* = 4). (**b**) LSS showed the greatest downregulation of *SIRT1*, followed by HSS and MSS (*n* = 4). (**c**) MSS showed the greatest upregulation of *EDN1*, followed by LSS and HSS (*n* = 4).

The expression of genes, relative to the housekeeping gene *GAPDH*, is presented as fold changes from the unstimulated control. The expression of *NOS3* was downregulated in all the ESS intensities compared to the unstimulated control; HSS showed the greatest downregulation (−6.06-fold) followed by LSS (−4.21-fold), MSS (−2.15-fold), and rest SS (−0.52-fold) (Figure 1a). The expression of *SIRT1* was upregulated in response to resting shear stress (0.90-fold), and was decreased with the other ESS intensities compared to the unstimulated control; LSS showed the greatest decrease (−3.13-fold) followed by HSS (−2.93-fold) and MSS (−2.67-fold) (Figure 1b). The expression of *EDN1* was upregulated in all ESS intensities compared to the unstimulated control; and MSS showed the greatest increase (2.89-fold), followed by LSS (2.20-fold), HSS (1.71-Fold), and rest ESS (1.68-fold) (Figure 1c).

### 3.2. Protein Expression

**Figure 2:**
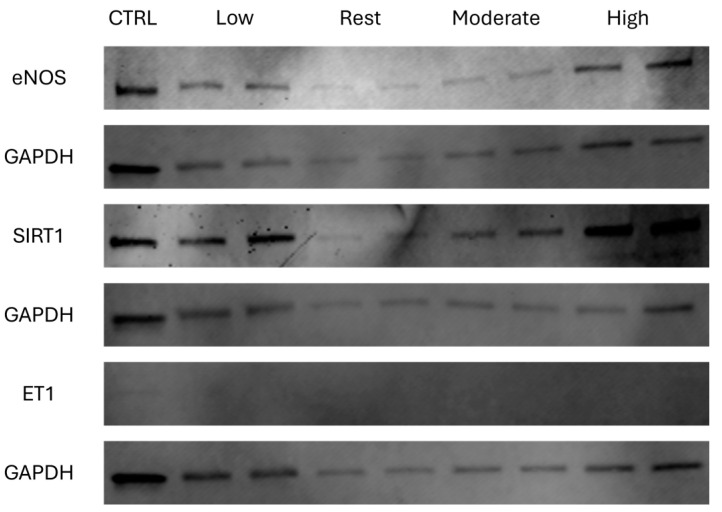
Western blot bands of target proteins eNOS, SIRT1, and ET1 with the corresponding bands of the loading control GAPDH (*n* = 4).

**Figure 3:**
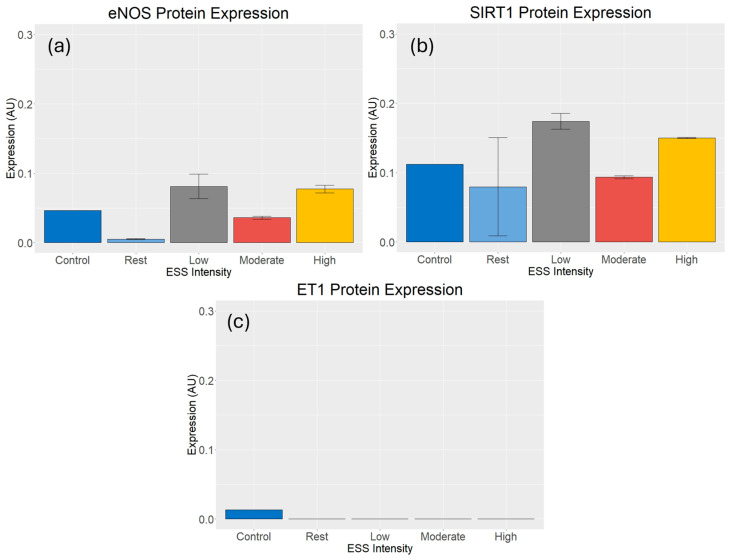
Protein expression after exercise-induced ESS. (**a**) LSS showed the highest expression of eNOS, followed by HSS and MSS, compared to resting conditions (*n* = 4). (**b**) LSS showed the highest expression of SIRT1, followed by HSS and MSS, compared to resting conditions (*n* = 4). (**c**) There was no expression of ET1 for any of the conditions, including rest (*n* = 4).

The fluorescence intensity of immunoblots was quantified (AU) to assess the changes in eNOS, SIRT1, and ET1 after HUVECs were exposed to different ESS intensities (Figure 2). There was a higher, non-statistically significant (*p* = 0.126) expression of eNOS with all exercise conditions compared to rest; LSS showed the highest expression (342.41 AU), followed by HSS (335.73 AU) and MSS (155.70 AU) (Figure 3a). Similarly to eNOS, there was a higher, non-statistically significant (*p* = 0.204) expression of SIRT1 with all exercise conditions compared to rest; LSS showed the highest expression (702.49 AU), followed by HSS (615.75 AU) and MSS (383.45 AU) (Figure 3b). There was no expression of ET1 in any of the conditions including rest, and no statistically significant difference (*p* = 0.92) between conditions (Figure 3c). Western blot images represent the average and standard error of the technical duplicates.

### 3.3. Correlations between Gene and Protein Expression

**Figure 4:**
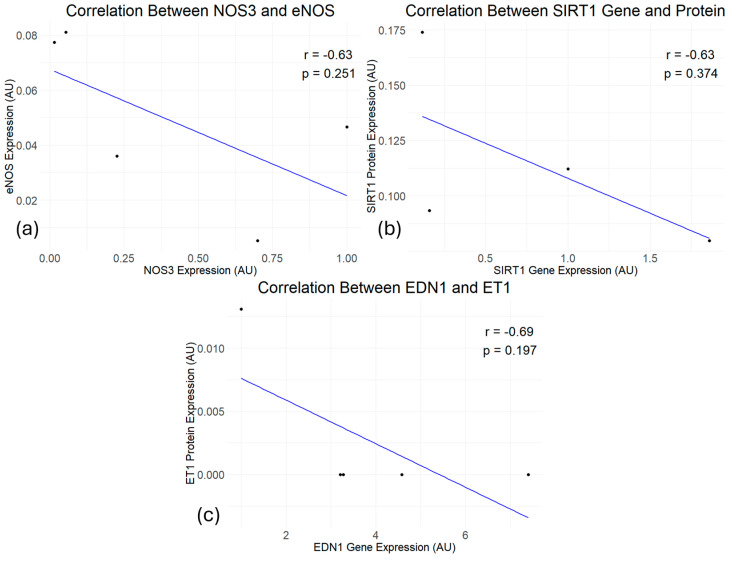
Correlation between gene and protein expression (*n* = 4). (**a**) non-significant negative correlation between *NOS3* and eNOS (*n* = 4). (**b**) Non-significant negative correlation between the *SIRT1* gene and the SIRT1 protein (*n* = 4). (**c**) Non-significant negative correlation between *EDN1* and ET1 (*n* = 4).

The analysis of the correlation between the gene and protein expressions resulted in non-significant (*p =* 0.05) negative correlations for all the genes. The correlation between *NOS3* and eNOS showed a non-significant negative correlation (r = −0.63, *p =* 0.251) (Figure 4a). Similar to the correlation between *NOS3* and eNOS, the correlation between the *SIRT1* gene and the SIRT1 protein showed a non-significant negative correlation (r = −0.63, *p =* 0.374) (Figure 4b). The correlation between *EDN1* and ET1 also showed a non-significant negative correlation (r = −0.69, *p =* 0.197) (Figure 4c).

## 4. Discussion

The proper function and maintenance of the endothelium are key in preventing CVDs [34]. The activity of the key molecules eNOS, SIRT1, and ET1 regulates endothelial function and vascular tone [23,35,36,37]. The changes in ESS have been shown to have a direct impact in the expression of these molecules [37,38]. In general, an increase in shear stress helps to protect the endothelium by increasing eNOS and SIRT1 availability and activity, and decreasing ET1 [39,40,41].

Physical activity, especially endurance exercise, increases blood flow, creating an exercise-induced ESS [19]. In general, the increase in exercise-induced ESS improves endothelial function and helps to prevent CVDs [18,42]. However, high-intensity exercise-induced ESS may be detrimental to endothelial health. Research suggests that very-high-intensity exercise may cause cardiovascular damage [43,44]. Therefore, the purpose of this research was to assess the effects of different exercise-induced ESS intensities on the expression of the proteins and their associated genes that are responsible for maintaining vascular health.

The results of our study show an overall, non-statistically significant increase in proteins eNOS and SIRT1 with a corresponding downregulation of their genes in all ESS intensities. No expression of ET1 with a corresponding upregulation of its gene was observed in any ESS intensity. Also, we show a non-statistically significant negative correlations between the gene and protein expression of all targets. These results show that (1) exercise-induced ESS increases the expressions of vascular protective proteins and (2) the relationship between protein and gene expression suggests that exercise could act as a regulator of mRNA translation to protein, regardless of ESS intensity.

Our results showed a downregulation of *NOS3* with a concomitant increase in eNOS, indicating enhanced NO production. This increase in NO, associated with exercise-induced shear stress and an increase in eNOS bioavailability, may lead to improvements in endothelial function by increasing vasodilation and blood flow [45], to potentially promote angiogenesis and vascular remodeling [46,47]. The associated increase in NO can potentially protect endothelial cells from oxidative stress-induced apoptosis, further improving cardiovascular health [48,49]. Similar to *NOS3* and eNOS, we showed a downregulation of the *SIRT1* gene and SIRT1 protein expressions, leading to an increase in NO by deacetylating and activating eNOS [36,50]. Additionally, SIRT1 is associated with the regulation of the inflammatory response, acting as an anti-inflammatory agent by inhibiting the NF-kB pathway [51,52], further protecting the endothelial cells from oxidative stress and inflammation. Finally, we showed an upregulation of *EDN1* and no expression of ET1, which are the key mediators of vasoconstriction. This reduction in vasoconstriction is directly associated with the increase in vasodilation caused by increases in eNOS and SIRT1, favoring the increase in vasodilation and improving blood flow [37]. This reduction in ET1 that is associated with exercise-induced ESS may contribute to a reduction in vascular inflammation and increase angiogenesis, minimizing the negative effects on vascular health [53].

In molecular biology, the central dogma states that DNA transcribes to mRNA, and mRNA translates to proteins [54]. In an unchanged environment, it is suggested that mRNA and its associated protein have a one-to-one relationship [55]. However, environmental changes can cause disturbances in this one-to-one association, and we can see negative relationships [55,56]. In our study, the exposure to low- and high-intensity exercise-induced ESS altered the cell’s environment, significantly upregulating the expression of eNOS and SIRT1, and downregulating the expression of their corresponding genes. At the same time, exercise-induced ESS downregulated the expression of ET1 and its corresponding gene in low-, moderate-, and high-intensity exercise-induced ESS.

This negative relationship shown in our results can be explained by considering the type of mRNA and the protein translation site. In mammalian cells, >80% to 90% of mRNA is present in ribosomes (>80% to 90%), where it is translated into proteins [57,58]. In our study, we extracted total mRNA, suggesting that most of the mRNA used in our experiments was ribosomal mRNA. Following biology’s central dogma, it is possible that exercise-induced ESS acted as a catalyst in the translation of mRNA to protein [59], signaling the immediate need to increase the production of eNOS and SIRT1, decrease the production of ET1 by translating *NOS3* and *SIRT1*, and stopping the translation of *EDN1* in the ribosomes to meet the demands of increased ESS [60,61].

It can be argued that the decrease in the genes may be due to the natural degradation of mRNA. However, it has been reported that even though mRNA is generally regarded as being very unstable and degrading fast, the half-life of mRNA ranges from 1 h up to 24 h, depending on the number of exons [62,63]. In our study, all the mRNA samples were extracted immediately after the end of exercise-induced ESS, and the time between cessation of exposure and extraction was less than 5 min [63]. Therefore, even considering the lowest reported mRNA half-life of about 1 h, our samples were not significantly affected by degradation. Also, the target genes had more than 5 exons, placing them in a group of genes with a half-life of at least 2 h [64,65,66].

No experiment is free of limitations, and our study is no exception. We were limited to replicating arterial ESS intensities that were previously measured by our group in a cell culture model [6,31,32], which lacks the complex interaction of environmental factors that could affect the expression of genes and proteins. We used HUVECs without specific information about the sex of the donor, which may have affected the expression of eNOS, and maintained the intensity of ESS for the duration of the experiment, which is rarely the case during exercise due to breaks during exercise, physiological adjustments, and environmental factors, especially during high-intensity exercise [67,68]. Also, some cells may detach from the slides due to the shear forces, resulting in variations in protein concentration, as shown in the Western blot image (Figure 2).

With the current knowledge of the acute interactions between exercise-induced ESS and the expression of genes and proteins, it will be interesting to include additional genes that are associated with endothelial cell function, including *VGEFA*, *VCAM1*, and *ICAM*. Future studies should also explore if exercise-induced ESS is an acute translation catalyst in a population of endurance-trained participants. This population has been shown to have an elevated expression of eNOS and SIRT1 with their corresponding genes [29,69,70]. Also, it will be interesting to explore if lactate produced during exercise influences translation during exercise.

## 5. Conclusions

In conclusion, exercise-induced ESS improves the expression of proteins associated with endothelial health by translating their associated genes, acting as an acute catalyst regardless of ESS intensity.

## Data Availability

The dataset and script used in this study can be found in online repositories. The files can be accessed on https://github.com/daconde7/Exercise-Induced-Shear-Stress-Drives-mRNA-Translation-in-vitro.git (accessed on 31 August 2024).
